# Morphological Variation and Integration of the Ethmoidal and Prechiasmatic Regions in Sheep

**DOI:** 10.3390/ani16071098

**Published:** 2026-04-02

**Authors:** Eylem Bektaş Bilgiç, Barış Can Güzel, Fatma İşbilir, Aycan Korkmazcan, Yusuf Altundağ, Nedžad Hadžiomerović, Ozan Gündemir

**Affiliations:** 1 Department of Surgery, Faculty of Veterinary Medicine, Istanbul University-Cerrahpasa, Istanbul 34320, Türkiye; eylem.bilgic@iuc.edu.tr; 2 Department of Anatomy, Faculty of Veterinary Medicine, Siirt University, Siirt 56100, Türkiye; baris.guzel@siirt.edu.tr (B.C.G.); fatma.isbilir@siirt.edu.tr (F.İ.); 3 Institute of Graduate Studies, Istanbul University-Cerrahpasa, Istanbul 34320, Türkiye; aycan.korkmazcan@ogr.iuc.edu.tr; 4 Department of Surgery, Faculty of Veterinary Medicine, Namik Kemal University, Tekirdag 59030, Türkiye; yaltundag@nku.edu.tr; 5 Department of Basic Sciences of Veterinary Medicine, Veterinary Faculty, University of Sarajevo, 71000 Sarajevo, Bosnia and Herzegovina; nedzad.hadziomerovic@vfs.unsa.ba; 6 Department of Anatomy, Faculty of Veterinary Medicine, Istanbul University-Cerrahpasa, Istanbul 34320, Türkiye; 7 Osteoarchaeology Practice and Research Centre, Istanbul University-Cerrahpasa, Istanbul 34320, Türkiye

**Keywords:** allometry, cranial base, endocranium, geometric morphometrics, shape analysis, veterinary anatomy

## Abstract

This study investigated breed- and sex-related variation in two adjacent regions of the sheep cranial base on the endocranial surface, namely the ethmoidal region (fossa ethmoidalis–crista galli complex) and the prechiasmatic region centered on the sulcus chiasmatis. We hypothesized that breed-related morphometric differences would be detectable in this cranial base region and that the ethmoidal and prechiasmatic regions would show modular separation despite substantial covariation. Three-dimensional landmark data from 113 adult Akkaraman, Morkaraman, and Zom sheep were analyzed. Whole-configuration size differed by breed and sex, ethmoidal size mainly by sex, and prechiasmatic size mainly by breed. Shape analyses showed significant breed effects in the whole configuration and in both regional modules, whereas sex effects were weaker and limited to the whole configuration and prechiasmatic region. Allometry was significant at all levels and was strongest in the two regional modules, especially the prechiasmatic region. Modularity analysis supported the separation of the ethmoidal and prechiasmatic landmarks into two modules, whereas integration analysis indicated a high degree of covariation between them (r-PLS = 0.933), with most shared covariance concentrated on the first PLS axis. These findings indicate that variation in this endocranial cranial base region is detectable in both size and shape, with shape differences identified more consistently across the whole configuration and the two regional modules, although the associated effect sizes were modest. Overall, the results highlight that variation between adjacent cranial base regions is primarily expressed through shape and covariation rather than size alone, providing a morphometric framework for future studies of cranial structure.

## Simple Summary

This study examined whether two neighboring areas on the inner surface of the skull base differ in three native sheep breeds. These areas are difficult to study using traditional methods, but imaging techniques allowed us to examine them in detail. We found differences between breeds in both size and shape, although the differences in shape were more noticeable. The two regions were partly independent but also changed together in a coordinated way. In contrast, their sizes were not strongly related. Overall, the results show that subtle differences between breeds can be detected even in hidden parts of the skull. They also suggest that nearby areas of the skull base are connected in how they vary, which may be useful for future studies of skull structure and development.

## 1. Introduction

The rostral cranial base on the endocranial surface of the cranium is an anatomically critical region formed mainly by the ethmoid and presphenoid bones [1,2]. In its rostral part lie the paired ethmoidal fossae, corresponding to the lamina cribrosa of the ethmoid bone, which are separated by the median projection of the crista galli. Because the fila olfactoria pass through the numerous foramina of the lamina cribrosa, the fossa ethmoidalis–crista galli complex constitutes the morphological core of the anterior cranial base associated with the olfactory compartment [1,3,4]. Immediately caudal to this region, on the body of the presphenoid bone, lies the sulcus chiasmatis, which is situated rostral to the hypophyseal fossa, continues laterally toward the optic canal, and forms a bony bed for the optic chiasm [5,6]. Accordingly, these two adjacent regions form an anatomically contiguous portion of the rostral cranial base, associated respectively with the ethmoidal compartment and the prechiasmatic area surrounding the optic chiasm [7].

Because the fossa ethmoidalis–crista galli complex and the sulcus chiasmatis region are located on the internal surface of the cranial base, direct anatomical access in dry skulls is limited and may require removal or damage of surrounding osseous structures [8,9]. Computed tomography offers a major advantage in this context by allowing non-destructive, multiplanar visualization of mineralized anatomy and enabling digital segmentation and three-dimensional reconstruction of otherwise difficult-to-access skull-base regions. For this reason, CT-based reconstruction has become an increasingly valuable tool in contemporary anatomical and morphometric studies, including veterinary investigations of cranial morphology and the three-dimensional documentation of complex osseous structures [10,11,12,13].

Geometric morphometrics provides a particularly suitable framework for the analysis of complex cranial regions because it captures morphology through the Cartesian coordinates of homologous landmarks rather than through isolated linear distances alone [14,15]. Following Procrustes superimposition, landmark configurations can be used to separate shape from the effects of position, orientation, and scale, thereby allowing the quantification and visualization of shape variation in morphospace, as well as the assessment of size-related shape change through allometric analyses [16,17]. Importantly, the same framework is also highly appropriate for studies of modularity and morphological integration, because it preserves the anatomical relationships among landmarks and permits explicit testing of whether adjacent regions behave as relatively independent modules or as strongly covarying parts of a single functional complex [18,19].

Previous studies in sheep have documented cranial variation mainly at the level of overall skull morphology, external cranial traits, or radiological description in different breeds [13]. These studies support the presence of breed-related cranial differences, but they do not clarify whether comparable variation is also expressed on the endocranial surface of the rostral cranial base. In particular, the internal morphology of the ethmoidal and prechiasmatic regions has not, to our knowledge, been quantitatively evaluated in sheep using landmark-based three-dimensional geometric morphometrics.

The focus on the ethmoidal and prechiasmatic regions is relevant for both anatomical and comparative reasons. These two subregions occupy adjacent positions on the rostral cranial base, can be identified reproducibly on CT-derived endocranial reconstructions, and represent anatomically distinguishable parts of the same cranial base scaffold. Their adjacency makes them suitable for testing whether breed-related variation is distributed uniformly across this region or partitioned between subregions, whereas their anatomical distinctness makes them appropriate for evaluating modularity and morphological integration within a shared structural context. The selected breeds (Akkaraman, Morkaraman, and Zom) were included based on their availability within the osteological collection, representing commonly studied native sheep breeds with documented variation in cranial morphology. Accordingly, the present study examined the ethmoidal and prechiasmatic regions in Akkaraman, Morkaraman, and Zom sheep using three-dimensional geometric morphometric methods. We hypothesized that breed-related differences would be detectable in both size and shape, that allometry would contribute to observed shape variation, and that the two regions would show modular separation despite strong covariation.

## 2. Materials and Methods

### 2.1. Study Sample and Osteological Collection

The cranial specimens used in this study were obtained from the osteological collection of the Istanbul University–Cerrahpasa, Osteoarchaeology Application and Research Center. According to the collection records, all individuals included in the study were older than 1 year, and sex information was available for every specimen. A total of 113 individuals were examined, representing three breeds: Akkaraman (n = 36; 13 females, 23 males), Morkaraman (n = 40; 20 females, 20 males), and Zom (n = 37; 16 females, 21 males). Restricting the analysis to adult specimens was intended to reduce the effect of ontogenetic variation on cranial form.

According to the collection records, all individuals included in the study were older than 1 year, and sex information was available for every specimen. Because exact chronological ages were not available beyond this classification, adult status was defined on the basis of collection records, and residual within-adult ontogenetic variation cannot be excluded. In addition, detailed metadata regarding geographic origin, flock management conditions, and population structure were not available for all specimens and therefore could not be incorporated into the analytical design. For this reason, the breed factor used in the present study should be interpreted as a collection-based grouping variable reflecting breed assignment in the osteological records rather than as a pure estimate of breed-specific genetic effects. Accordingly, breed assignment in the present study is based on collection records and should be interpreted as a grouping variable rather than a direct measure of breed-specific genetic or adaptive differences.

### 2.2. CT Imaging and 3D Modelling

Segmentation and three-dimensional reconstruction were performed in the Segment Editor module of 3D Slicer (version 5.2.2) using a standardized semi-manual workflow [20]. The target cranial base region was isolated by multiplanar slice-by-slice editing in the axial, sagittal, and coronal views, with consistent visual criteria applied across specimens to preserve the anatomical margins used for landmark placement. Non-target fragments were removed during reconstruction, and each model was reviewed in both multiplanar and three-dimensional views before landmark digitization. Because exact numeric segmentation thresholds were not retained as part of the study records, they cannot be reported retrospectively; however, the same reconstruction workflow was used for all specimens. Because the scans were acquired from isolated dry skull specimens, motion-related artifacts were not expected. In addition, all image series were visually screened before and during segmentation for streaking, beam-hardening, discontinuities, or other artifacts affecting the target region. A formal external validation of geometric accuracy against direct osteometric measurements or a calibration phantom was not performed. Accordingly, the present study should be interpreted as a standardized comparative morphometric analysis rather than as a validation study of reconstruction fidelity. Segmentation was guided by visual differentiation between mineralized bone and surrounding structures, with thresholding used only as an initial step and subsequent manual refinement applied to preserve anatomical boundaries relevant to landmark placement.

### 2.3. Landmarking

**Figure 1:**
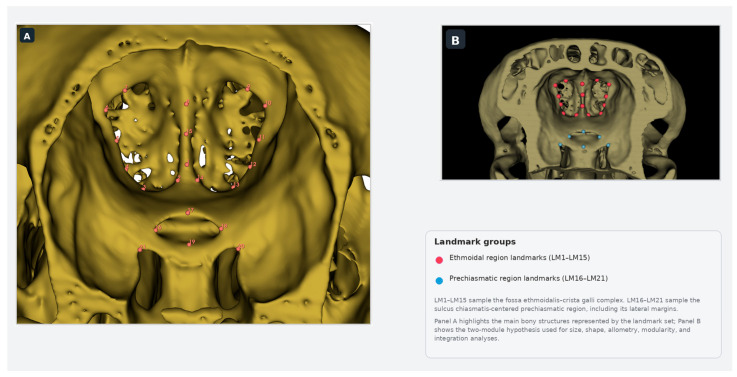
Landmark configuration and a priori module partition on the endocranial surface of the sheep cranial base. (**A**) Numbered landmark configuration (LM1–LM21) digitized from CT-derived three-dimensional reconstructions. LM1–LM15 sample the fossa ethmoidalis–crista galli complex, whereas LM16–LM21 sample the sulcus chiasmatis-centered prechiasmatic region and its lateral margins. Major anatomical references shown in Panel (**A**) include the paired ethmoidal fossae, crista galli, and sulcus chiasmatis. (**B**) A priori two-module partition used in downstream size, shape, allometry, modularity, and integration analyses.

After three-dimensional reconstruction, landmark data were collected by manual digitization in 3D Slicer (v5.2.2). A total of 21 three-dimensional anatomical landmarks were placed on each specimen to capture the geometry of the ethmoidal and prechiasmatic region (Figure 1, Table A1). Landmarks were digitized in a standardized endocranial orientation so that homologous points could be identified consistently across all individuals. The first 15 landmarks (LM1–LM15) represented the fossa ethmoidalis–crista galli complex, whereas LM16–LM21 represented the sulcus chiasmatis-centered prechiasmatic region and its lateral margins used in the module-based analyses. The unequal number of landmarks reflects the greater anatomical extent and landmark richness of the ethmoidal region relative to the more restricted prechiasmatic region, while preserving only reproducible osteological reference points in both modules. All landmarking was performed manually using the same configuration for every specimen, producing a comparable coordinate dataset for subsequent geometric morphometric analyses. Detailed definitions of all landmarks used in the study are provided in Appendix A (Table A1).

To assess intraobserver repeatability, the same researcher digitized the landmark configuration twice, and the replicated datasets were compared using Procrustes ANOVA. The measurement error effect was not significant (*p* > 0.05), indicating that repeated landmark placement did not introduce detectable shape variation. This repeatability assessment pertains to landmark digitization consistency and should not be interpreted as an external validation of CT reconstruction accuracy. Landmark placement was performed without reference to breed or sex labels to minimize potential observer bias during digitization.

### 2.4. Statistical Analyses

All statistical analyses were carried out in R (version 4.0.5) [21]. Specimen labels were parsed to assign individuals to breed (Akkaraman, Morkaraman, Zom) and sex (female, male). Landmark configurations were subjected to Generalized Procrustes Analysis (GPA) to remove the effects of translation, rotation, and scale, and the resulting Procrustes coordinates were used for subsequent shape analyses, including principal component analysis (PCA).

For size analyses, the centroid size (CS) value associated with each configuration was used as the global size variable. To evaluate regional size variation, landmark subsets corresponding to the ethmoidal region and the prechiasmatic region were extracted from the configuration, and centroid size was calculated separately for each subset. These values were used as module-level size descriptors for the ethmoidal and prechiasmatic regions.

Differences in size among breeds and sexes were evaluated separately for the whole configuration, the fossa ethmoidalis–crista galli complex, and the sulcus chiasmatis-centered prechiasmatic region using linear models with the form log (CS) ~ breed × sex. Estimated marginal means were obtained for the breed-by-sex combinations, and post hoc pairwise comparisons were adjusted using Tukey procedures. The relationship between fossa ethmoidalis–crista galli complex size and sulcus chiasmatis-centered prechiasmatic region size was further examined using linear regression with breed and sex included as interaction terms. Boxplots were generated for the three size variables, and bivariate scatterplots were displayed with group-specific convex hulls. When omnibus effects were significant, post hoc pairwise comparisons were used to clarify the direction of group differences and are summarized in Section 3.

For shape analyses, Procrustes shape coordinates were analyzed in the geomorph (v.4.0.4) framework [22,23]. using Procrustes ANOVA implemented through residual randomization permutation procedures (RRPP) [24]. The primary model tested was shape ~ breed × sex, and separate analyses were performed for the whole landmark configuration, the fossa ethmoidalis–crista galli complex, and the sulcus chiasmatis-centered prechiasmatic region. Significance was evaluated from 9999 permutations. Where appropriate, pairwise group differences were summarized from the RRPP output. Morphological variation in shape space was then explored using principal component analysis (PCA) of the Procrustes coordinates. PC1–PC2 scatterplots were used descriptively to visualize group dispersion, and convex hulls were added to show the extent of morphospace occupation by each breed-by-sex group. For graphical interpretation, the negative and positive shape changes associated with the principal axes were visualized by displacing the consensus configuration along PC1 and PC2.

Allometry was assessed separately for the whole configuration and for each landmark subset by fitting Procrustes models that included log-transformed size as a covariate (shape ~ log size × breed × sex). In addition to the permutation-based model tests, allometric scores derived from the regression of shape on size were used for visualization, and scatterplots were displayed with group-specific convex hulls. Finally, a two-module hypothesis was evaluated in which LM1–LM15 represented the fossa ethmoidalis–crista galli complex and LM16–LM21 represented the sulcus chiasmatis-centered prechiasmatic region. This partition was defined a priori based on anatomical and regional criteria, rather than through data-driven module detection. Modularity was tested using the covariance ratio (CR) framework [25], whereas integration between the two landmark blocks was evaluated using PLS-based covariation tests [24,26]. PLS1 scores of the two partitions were plotted to summarize the main axis of covariation between the ethmoidal and prechiasmatic region.

In addition to statistical significance, effect sizes (e.g., R^2^ values and covariance measures) were considered when interpreting the magnitude of observed differences.

## 3. Results

### 3.1. Sample Composition and Centroid Size Summary

The dataset comprised 113 adult specimens: 36 Akkaraman, 40 Morkaraman, and 37 Zom sheep, including 49 females and 64 males. As shown in Table 1, Zom sheep showed the highest centroid size values. The descriptive values in Table 1 are presented in the original centroid-size scale.

**Figure 2:**
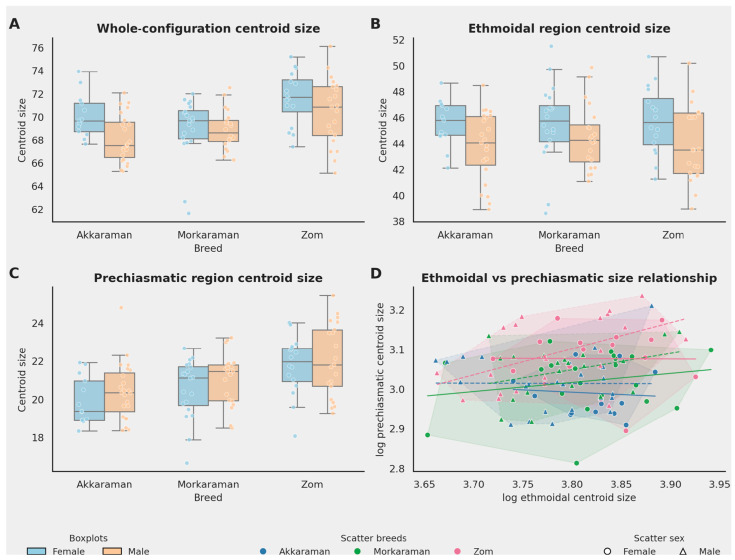
Composite size figure. Panels (**A**–**C**) show grouped boxplots for the whole configuration, ethmoidal region, and prechiasmatic region centroid size, respectively. Panel (**D**) shows the relationship between ethmoidal and prechiasmatic size by breed and sex; convex hulls are shown for the six breed-by-sex groups.

Size analyses indicated that the magnitude of variation differed by configuration (Table 2). Whole-configuration centroid size differed significantly among breeds and between sexes, with no significant breed × sex interaction. Ethmoidal region size was influenced mainly by sex, whereas prechiasmatic region size was influenced mainly by breed. These patterns are visualized in Figure 2A–C, where Zom animals, especially in the prechiasmatic region, generally occupied the upper portion of the size range.

The size relationship between the two regional modules was weak and not statistically significant (r = 0.159, *p* = 0.093), indicating that variation in ethmoidal and prechiasmatic centroid size was largely independent at the global level. Although some within-group patterns were observed (e.g., in Zom males), these did not alter the overall weak association between the two regions.

### 3.2. Shape Results

**Figure 3:**
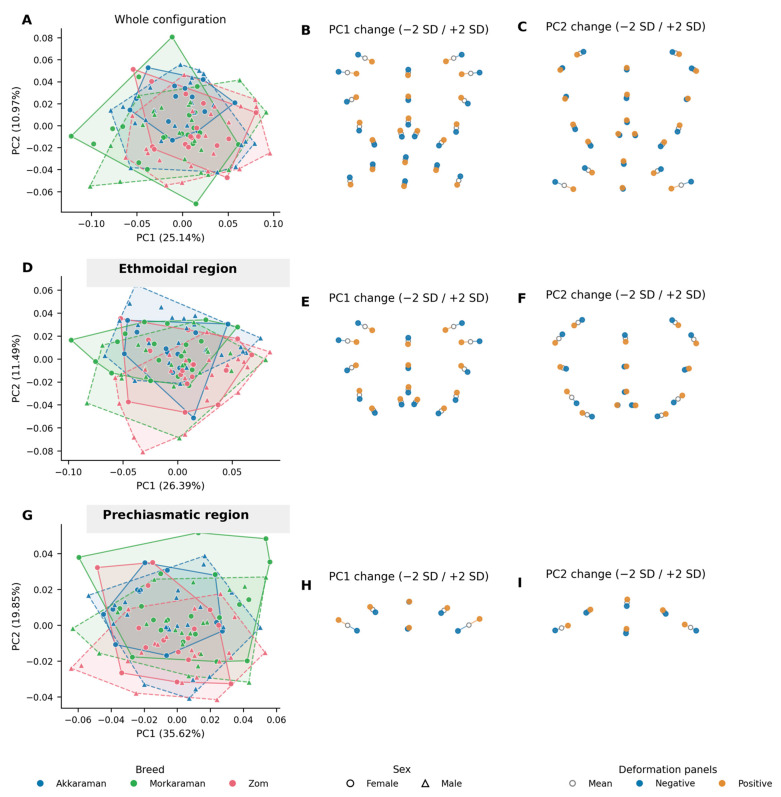
Principal component shape variation with negative and positive changes along PC1 and PC2. The left column shows PCA scatterplots with convex hulls for the breed-by-sex groups. The middle and right columns show landmark displacement patterns in XY projection at −2 SD and +2 SD from the mean shape along PC1 and PC2, respectively (gray = mean; blue = negative change; orange = positive change). Positive and negative directions along the axes represent shape changes relative to the mean configuration. Overlap between groups indicates that differences are expressed as directional trends rather than fully discrete separation. Subfigures (**A**–**C**) represent the whole configuration, where (**A**) shows the PCA distribution with group overlap, (**B**) illustrates shape changes along PC1 (−2 SD/+2 SD), and (**C**) shows shape changes along PC2 (−2 SD/+2 SD). Subfigures (**D**–**F**) correspond to the ethmoidal region, including PCA distribution (**D**), PC1 variation (**E**), and PC2 variation (**F**). Subfigures (**G**–**I**) represent the prechiasmatic region, where (**G**) shows the PCA distribution, (**H**) illustrates PC1 changes, and (**I**) shows PC2 changes.

Permutation-based Procrustes ANOVA showed a significant breed effect for the whole configuration, the ethmoidal region, and the prechiasmatic region (Table 3). Sex also affected overall shape and prechiasmatic shape, whereas the sex effect for the ethmoidal region did not reach significance. No breed × sex interaction was detected in any shape model, indicating that breed-related shape differences were not strongly sex-specific. These multivariate effects are consistent with the PCA scatterplots in Figure 3A,D,G, where the breed groups show partial displacement in morphospace but still retain substantial overlap.

The first two principal components accounted for 36.11% of total shape variance in the whole configuration, 37.88% in the ethmoidal region, and 55.47% in the prechiasmatic region (Figure 3). In the ethmoidal region, PC1 (Figure 3E) contrasted a relatively laterally expanded, broader arrangement of the paired ethmoidal fossae at negative scores with a narrower and more compact fossa ethmoidalis–crista galli configuration at positive scores. PC2 (Figure 3F) mainly reflected a secondary dorsoventral and transverse reorganization, with positive scores corresponding to a slightly broader, lower arrangement and negative scores to a relatively taller, narrower pattern in the XY projection. In the prechiasmatic region, PC1 (Figure 3H) primarily described transverse widening of the sulcus chiasmatis-centered configuration, whereas PC2 (Figure 3I) represented a secondary mediolateral redistribution of the lateral prechiasmatic margins.

Breed tendencies within morphospace were directional rather than sharply discrete. In the whole-configuration and ethmoidal analyses, Zom individuals tended to occupy more positive PC1 values, whereas Morkaraman tended toward more negative PC1 values; Akkaraman showed relatively higher mean PC2 values in the same spaces. In the prechiasmatic PCA, Morkaraman tended toward more positive PC1 values, Akkaraman toward more negative PC1 values, and Zom toward lower PC2 values. However, because the convex hulls overlapped broadly in Figure 3 and permutation-based pairwise mean-shape distances were not significant, these patterns reflect generalized breed trends rather than discrete morphotypes. Despite statistically significant effects detected in the Procrustes ANOVA, the substantial overlap in morphospace and lack of significant pairwise differences indicate that these patterns reflect partial and directional variation rather than clearly discrete morphological differentiation among breeds.

### 3.3. Allometry, Modularity, and Integration

**Figure 4:**
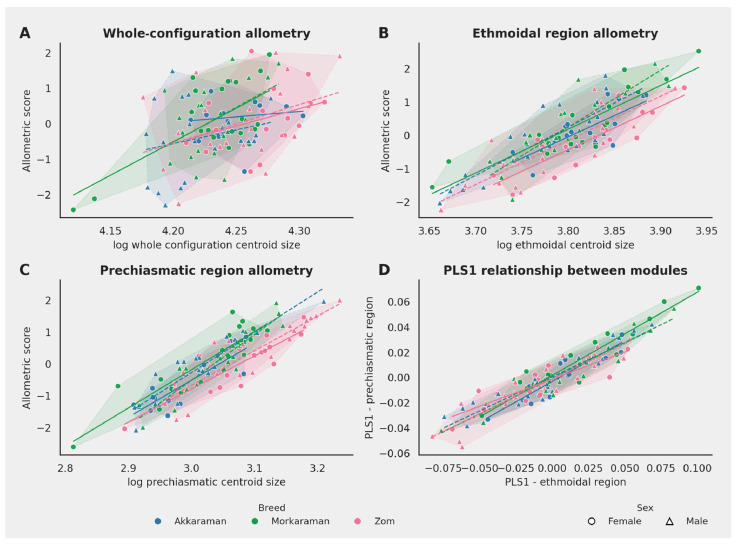
Allometry and covariation between the ethmoidal and prechiasmatic modules. Panels (**A**–**C**) show allometric patterns for the whole configuration, ethmoidal region, and prechiasmatic region, respectively, with convex hulls for the six breed-by-sex groups. Panel (**D**) shows the PLS1 relationship between the two modules. The slope of the fitted lines represents the relationship between size and shape, while overlap among groups indicates similar allometric trends across breeds.

Allometry was significant for the whole configuration, the ethmoidal region, and the prechiasmatic region, as summarized in Table 4. The larger R^2^ values for the two regional modules indicate that size-related shape change was more clearly expressed within the ethmoidal and prechiasmatic regions than in the whole configuration, although the proportion of explained variance remained moderate overall. Figure 4A–C visually supports this interpretation: the fitted trends are shallowest in the whole configuration and clearly steeper in the two regional modules, especially in the prechiasmatic region.

None of the size × breed, size × sex, or size × breed × sex interaction terms reached significance (Table 4), indicating that the main allometric trajectories were broadly parallel across breeds and sexes rather than group-specific. This suggests that breed-related shape differences were suggesting that group differences were expressed along broadly similar allometric trends rather than through distinct scaling relationships, although the relatively low proportions of explained variance indicate that size accounts for only a limited part of overall shape variation. The hull structure in Figure 4A–C likewise shows extensive overlap among the six groups despite detectable differences in trajectory position.

In contrast to the weak size association observed between the two regions, shape-based integration analysis revealed very strong covariation. The complementary modularity and integration analyses are summarized in Table 5. The modularity test supported a significant two-module organization for the ethmoidal (LM1–LM15) and prechiasmatic (LM16–LM21) landmark blocks (CR = 0.844, *p* = 0.006), indicating that the two adjacent landmark-defined regions behaved as separable components of the cranial base. At the same time, the integration test revealed very strong covariation between the same modules.

Table 5 further shows that 80.90% of the shared covariance was concentrated on PLS1 and that the correlation between ethmoidal and prechiasmatic PLS1 scores was also very high. This combined pattern is visualized in Figure 3D, where the strong positive relationship between module scores demonstrates that variation in the ethmoidal region was accompanied by parallel variation in the adjacent prechiasmatic region. Taken together, Table 5 and Figure 4 indicate that the two regions were morphologically distinguishable as modules while remaining tightly linked in their covariation.

## 4. Discussion

The present study was designed to test whether the endocranial rostral cranial base region encompassing the ethmoidal and prechiasmatic regions shows breed- and sex-related morphometric variation in sheep and whether these adjacent regions behave as relatively independent or tightly coordinated anatomical units. Using a sample of 113 adult specimens, we identified significant breed-related effects in size and shape, detected allometric patterning, and demonstrated that the ethmoidal and prechiasmatic regions were both modular and strongly integrated. Taken together, these results indicate that breed-related morphometric variation is detectable in this endocranial cranial base region. Previous studies have demonstrated breed-related differences in other parts of the sheep skull; the present results extend this observation to the endocranial rostral cranial base [27,28,29].

The interpretation of the present results requires a distinction between different levels of inference. The analyses conducted here are based on osteological landmark configurations and therefore primarily provide descriptive and morphometric information on size, shape, allometry, modularity, and covariation. While these patterns may be discussed in a broader biological context, they do not directly demonstrate developmental mechanisms or functional differences. Accordingly, any references to biological relevance should be interpreted as contextual and hypothesis-generating rather than as direct evidence of functional or adaptive differentiation.

One of the clearest outcomes of the study is that size and shape did not follow exactly the same pattern. As shown in Table 2 and Figure 2, whole-configuration centroid size differed significantly by both breed and sex, ethmoidal size was influenced mainly by sex, and prechiasmatic size was influenced mainly by breed. Zom sheep tended to occupy the upper part of the size distribution, particularly for the whole configuration and the prechiasmatic region, whereas ethmoidal size showed more limited breed separation. This pattern suggests that breed-related differentiation in this part of the sheep cranial base is not simply a matter of overall enlargement or reduction. Rather, the most informative signal appears to lie in how the landmarks are spatially reorganized, which is why shape analyses proved more discriminative than centroid size alone.

An important distinction emerging from the present results is the difference between size-based and shape-based relationships. While the centroid size relationship between the ethmoidal and prechiasmatic regions was weak and not statistically significant, shape analyses revealed strong covariation between the same regions. This indicates that structural coordination between these parts of the cranial base is primarily expressed at the level of shape rather than absolute size.

Although allometric effects were statistically significant and consistent across analyses, the associated R^2^ values indicate that size explains only a limited proportion of total shape variation, particularly at the level of the whole configuration. Therefore, the observed allometric patterns should be interpreted as contributing to, but not dominating, overall morphological variation.

This interpretation is reinforced by the shape results. Table 3 and Figure 3 show that breed significantly affected the whole configuration, the ethmoidal region, and the prechiasmatic region, whereas sex effects were weaker and more localized. The absence of significant breed × sex interactions indicates that breed-related shape differentiation was broadly similar in females and males rather than being expressed through breed-specific sexual patterns. In practical terms, this means that breed identity left a detectable imprint on the endocranial cranial base, but that this imprint was expressed as a multivariate pattern shared across sexes rather than as sharply distinct sex-dependent morphotypes.

The principal component plots further clarify the nature of these shape trends. In the ethmoidal region, the main axis of variation described a transition from a more laterally expanded and broader arrangement of the paired ethmoidal fossae to a narrower and more compact fossa ethmoidalis–crista galli configuration, while the second axis reflected a secondary redistribution of height and width within the same complex. In the prechiasmatic region, the first axis mainly captured transverse widening of the sulcus chiasmatis-centered configuration, whereas the second axis represented finer mediolateral rearrangement of the lateral prechiasmatic margins. Breed tendencies in morphospace were therefore directional rather than discrete: Zom tended toward more positive PC1 values in the whole-configuration and ethmoidal analyses, Morkaraman tended toward more negative PC1 values in those same spaces, and Akkaraman showed relatively higher mean PC2 tendencies. In the prechiasmatic analysis, Morkaraman shifted more toward positive PC1 values, Akkaraman toward more negative PC1 values, and Zom toward lower PC2 values. However, because the convex hulls in Figure 3 still overlapped broadly and pairwise mean-shape distances were not significant, these trends should be interpreted as generalized breed tendencies rather than as fully separate morphotypes.

Although statistically significant breed effects were detected in shape analyses, these results should be interpreted with caution. The broad overlap observed in morphospace and the absence of significant pairwise mean-shape differences indicate that the observed patterns do not represent fully discrete morphological differentiation among breeds. Instead, they reflect directional tendencies and partial structuring of variation within a shared morphospace.

Sex-related variation deserves separate consideration. Although sex affected whole-configuration size and shape and also influenced prechiasmatic shape, the ethmoidal region showed its strongest sex effect in size rather than in shape. This combination suggests that sexual dimorphism in the endocranial cranial base may be region-specific. In the ethmoidal region, sex may be expressed more through scaling differences, whereas in the prechiasmatic region, sex-related effects extend more clearly into geometric reorganization. Even so, the smaller magnitude of sex effects relative to the breed term suggests that differences among the breed-designated groups accounted for more variation than sex in this dataset.

Perhaps the most informative morphometric result is the combined evidence for modularity and strong integration. Table 5 shows that the ethmoidal and prechiasmatic landmark blocks behaved as statistically separable modules, supporting their treatment as anatomically distinguishable subregions of the same cranial base framework. At the same time, the correlation structure between these modules was very strong at the statistical level (r-PLS = 0.933), although this does not necessarily imply direct biological coupling. In addition, most of the shared covariance was concentrated on the first PLS axis, indicating that the dominant pattern of covariation between the two regions was highly organized rather than diffuse. Taken together, these results suggest that the two regions are morphologically distinguishable without being developmentally or spatially disconnected.

The coexistence of significant modularity and very strong integration is not contradictory but theoretically expected. Klingenberg’s work has emphasized that modules are not absolutely independent structures; rather, they are internally cohesive units that remain linked to other regions through shared development and growth [18,19]. Our results fit that framework closely: the ethmoidal and prechiasmatic landmark sets were statistically separable as modules, yet their covariation was concentrated along a strong first PLS axis. This supports the interpretation that the two regions are semi-distinct parts of a shared cranial base scaffold rather than unrelated skeletal patches [18,19,30]. The modular partition used in this study was defined a priori based on anatomical criteria, distinguishing the ethmoidal and prechiasmatic regions as two adjacent but structurally distinct parts of the rostral cranial base. Alternative partitioning schemes were not explored, as the primary aim was to test this specific biologically motivated hypothesis rather than to identify modules through data-driven approaches. Future studies may extend this framework by evaluating alternative modular configurations.

The strong integration observed between the two regions should also be interpreted with caution. Because the ethmoidal and prechiasmatic regions are anatomically adjacent and part of a continuous cranial base surface, some degree of covariation may reflect geometric proximity or shared spatial context rather than purely functional or developmental integration. In addition, the landmark distribution was unequal between the two modules (15 landmarks in the ethmoidal region versus 6 in the prechiasmatic region). Although this imbalance reflects underlying anatomical complexity and the limited number of reproducible landmarks available in the prechiasmatic region, it may also influence covariance structure, modularity estimates, and the apparent strength of integration. Therefore, while the results indicate strong statistical integration, the underlying sources of this covariation cannot be fully disentangled within the present study.

Breed-level interpretation should nevertheless remain cautious. Although long-term breed history may contribute to cranial differentiation, the present dataset does not allow this possibility to be separated from other unmeasured sources of variation, such as age heterogeneity within the adult sample, geographic background, management conditions, or population structure. At the same time, the functional literature suggests that olfaction in sheep is important for social recognition and contributes to early feed appraisal, whereas vision contributes to recognition, environmental monitoring, and learned visual discrimination [31,32,33]. These findings therefore support the presence of breed-related structural signals in neighboring olfactory-associated and visual-pathway-related endocranial regions, but they do not demonstrate breed differences in sensory performance per se. This limitation should be stated explicitly: because the present dataset is osteological, not neurofunctional, future studies should combine endocranial morphometrics with soft-tissue imaging, optic canal measurements, olfactory-bulb-related metrics, and behavioral data in order to determine whether the observed shape patterns reflect sensory ecology, general breed history, or both.

The observed morphometric differences among the three sheep breeds may reflect multiple underlying factors, but their interpretation should remain cautious. Differences in habitat, management conditions, or breed history could contribute to variation in cranial structure, including the endocranial base. In addition, because the ethmoidal and prechiasmatic regions are anatomically associated with olfactory and visual pathways, respectively, it is possible that some of the observed variation relates to differences in sensory-related organization. However, the present study is based solely on osteological data and does not directly assess sensory function, behaviour, or ecological adaptation. Therefore, such interpretations remain hypothetical and require further investigation using integrated anatomical, functional, and behavioural approaches.

Future research could extend these findings by incorporating soft-tissue imaging, functional analyses, and more detailed information on age, population structure, and environmental background. Such approaches would help clarify whether the morphometric patterns observed here are associated with functional, developmental, or ecological differences among breeds.

One limitation of the present study is the limited number of previous investigations focusing specifically on the endocranial cranial base region examined here. Because the ethmoidal and prechiasmatic regions have rarely been analyzed in sheep using geometric morphometric approaches, direct comparisons with earlier studies remain restricted. Consequently, the discussion of the present findings relies largely on broader cranial morphometric and anatomical literature rather than on region-specific precedents. Future studies examining similar endocranial structures in different sheep populations or related ruminant species would therefore be valuable for validating and expanding the patterns observed in the current dataset.

An additional limitation concerns sample metadata. Although all specimens were classified as adults according to collection records, and sex was known for all individuals, exact chronological age was not available beyond the threshold of older than 1 year. Therefore, some residual ontogenetic variation within the adult sample cannot be ruled out. Likewise, detailed information on geographic origin, flock management conditions, and population structure was not consistently available for all specimens. As a result, the observed differences among breed-designated groups should be interpreted cautiously as breed-associated morphometric patterns within this osteological collection rather than as unqualified breed effects attributable solely to breed history or genetic background. A further methodological limitation is that the geometric accuracy of the CT-derived three-dimensional reconstructions was not formally validated against an external reference standard such as direct osteometric measurements or a calibration phantom. Although image acquisition and reconstruction were standardized across specimens, and landmark digitization error was low, this does not substitute for formal validation of reconstruction fidelity. Consequently, subtle aspects of shape variation, covariance structure, and module-level comparisons should be interpreted with appropriate caution. Future studies should incorporate explicit geometric validation procedures together with fully reportable segmentation parameters.

## 5. Conclusions

In conclusion, the present study demonstrates that the endocranial cranial base of sheep exhibits measurable morphometric variation among the breed-designated groups examined here, with weaker and more localized effects of sex. Variation was detectable in both size and shape, with shape differences observed more consistently across the whole configuration and the two regional modules. The ethmoidal and prechiasmatic regions behaved as anatomically distinguishable yet strongly covarying parts of the same cranial base framework. Allometry was significant, particularly within the two regional modules, but its contribution to overall shape variation remained limited. Taken together, these results define the ethmoidal and prechiasmatic regions as a coordinated morphometric system within the sheep cranial base. These findings should be interpreted at the level of osteological and morphometric variation, as broader biological or functional implications remain tentative. Future research integrating morphometric, soft-tissue, and population-level data will be essential to better understand the biological significance of these patterns and their potential relevance for veterinary and comparative anatomy.

## Figures and Tables

**Table 1:** Sample sizes and centroid size means (±SD) by breed and sex.

Breed	Sex	N	Whole Configuration CS (Mean ± SD)	Ethmoidal Region CS (Mean ± SD)	Prechiasmatic Region CS (Mean ± SD)
Akkaraman	Female	13	70.06 ± 1.88	45.55 ± 1.81	19.91 ± 1.24
Akkaraman	Male	23	68.08 ± 2.05	43.77 ± 2.66	20.42 ± 1.50
Morkaraman	Female	20	68.96 ± 2.67	45.52 ± 3.04	20.55 ± 1.54
Morkaraman	Male	20	68.87 ± 1.70	44.47 ± 2.48	21.07 ± 1.37
Zom	Female	16	71.61 ± 2.32	45.74 ± 2.61	21.73 ± 1.56
Zom	Male	21	70.43 ± 2.88	44.04 ± 2.92	22.06 ± 1.90

**Table 2:** ANOVA summary for log-transformed centroid size variables.

Trait	Breed Effect (F, *p*)	Sex Effect (F, *p*)	Breed × Sex (F, *p*)	Model R^2^
Whole configuration CS	9.04, <0.001	5.87, 0.017	1.61, 0.204	0.207
Ethmoidal region CS	0.13, 0.882	8.79, 0.004	0.26, 0.774	0.085
Prechiasmatic region CS	10.79, <0.001	2.26, 0.136	0.07, 0.932	0.183

**Table 3:** Procrustes ANOVA summary for shape variation across the whole configuration and regional subsets.

Configuration	Breed (F, R^2^, *p*)	Sex (F, R^2^, *p*)	Breed × Sex (F, R^2^, *p*)	Configuration
Whole configuration	3.41, 0.058, <0.001	1.88, 0.016, 0.042	0.99, 0.017, 0.446	Whole configuration
Ethmoidal region	3.30, 0.056, <0.001	1.61, 0.014, 0.092	1.10, 0.019, 0.308	Ethmoidal region
Prechiasmatic region	3.71, 0.063, <0.001	2.57, 0.022, 0.029	0.70, 0.012, 0.730	Prechiasmatic region

**Table 4:** Allometric effects on shape variation based on permutation-based Procrustes ANOVA (9999 permutations).

Configuration	Size Effect (F, R^2^, *p*)	Size × Breed *p*	Size × Sex *p*	Size × Breed × Sex *p*
Whole configuration	2.06, 0.017, 0.028	0.245	0.635	0.235
Ethmoidal region	18.96, 0.130, <0.001	0.400	0.760	0.072
Prechiasmatic region	35.68, 0.220, <0.001	0.354	0.205	0.504

**Table 5:** Allometry summary from permutation-based Procrustes ANOVA (9999 permutations).

Analysis	Statistic	Effect/CI	*p* Value	Interpretation
Modularity test	CR = 0.8441	Z = −2.2838; 95% CI = 0.7762–0.9319	0.0060	Significant modular structure
Integration test	r-PLS = 0.9328	Z = 6.5395	0.0001	Strong integration between modules
PLS1 covariance contribution	80.90%	First singular axis	—	Most shared covariance concentrated on PLS1
PLS1 correlation	r = 0.9328	95% CI = 0.9038–0.9533	<0.0001	High covariation on the first PLS axis

## Data Availability

The data presented in this study are available upon request from the corresponding author (O.G.).

## Appendix A

### Landmark Definitions

**Table A1:** Definitions of the three-dimensional landmarks used for the geometric morphometric analysis of the ethmoidal and prechiasmatic regions.

Landmark(s)	Region	Proposed Landmark Name	Operational Definition
LM1	Ethmoidal	Ventral crista galli point	Ventral point of the crista galli on the endocranial surface, located at the lower part of the median ridge between the paired ethmoidal fossae.
LM2 and LM14	Ethmoidal	Caudomedial ethmoidal rim points	Most caudomedial points of the left and right ethmoidal fossae, respectively, located on the medial rim close to the base of the crista galli.
LM3 and LM13	Ethmoidal	Caudolateral ethmoidal rim points	Most caudolateral points of the left and right ethmoidal fossae, respectively.
LM4 and LM12	Ethmoidal	Ventrolateral ethmoidal rim points	Points located on the ventrolateral rim of the left and right ethmoidal fossae, respectively, at the level of the lower third of each fossa.
LM5 and LM11	Ethmoidal	Midlateral ethmoidal rim points	Points located on the lateral rim of the left and right ethmoidal fossae, respectively, at approximately mid-height.
LM6 and LM10	Ethmoidal	Dorsolateral ethmoidal rim points	Points located on the dorsolateral rim of the left and right ethmoidal fossae, respectively.
LM7 and LM9	Ethmoidal	Dorsomedial ethmoidal rim points	Points located on the dorsomedial rim of the left and right ethmoidal fossae, respectively, near the upper medial border of each fossa.
LM8	Ethmoidal	Dorsal crista galli point	Dorsal-most point of the crista galli visible on the endocranial surface.
LM15	Ethmoidal	Middle crista galli point	Midpoint of the crista galli on the endocranial surface, positioned between the dorsal and ventral crista galli landmarks.
LM16 and LM18	Prechiasmatic	Rostrolateral ends of the sulcus chiasmatis	Left and right rostrolateral extremities of the rostral lip of the sulcus chiasmatis, respectively.
LM17	Prechiasmatic	Rostral midpoint of the sulcus chiasmatis	Midpoint of the rostral lip of the sulcus chiasmatis.
LM19	Prechiasmatic	Caudal midpoint of the sulcus chiasmatis	Midpoint of the caudal lip/floor transition of the sulcus chiasmatis on the endocranial surface.
LM20 and LM21	Prechiasmatic	Lateral prechiasmatic groove points	Right and left rostral-lateral extremities of the lateral groove corresponding to the sulcus nn. ophthalmici et maxillaris, respectively.

Landmarks were defined using reproducible osteological margins, ridge extrema, and regional boundary points visible on the endocranial surface to maximize repeatability of manual landmark digitization.
